# No infectious SARS-CoV-2 in breast milk from a cohort of 110 lactating women

**DOI:** 10.1038/s41390-021-01902-y

**Published:** 2022-01-19

**Authors:** Paul Krogstad, Deisy Contreras, Hwee Ng, Nicole Tobin, Christina D. Chambers, Kerri Bertrand, Lars Bode, Grace M. Aldrovandi

**Affiliations:** 1 Department of Pediatrics, David Geffen Scool of Medicine at UCLAs, University of California, Los Angeles, CA USA; 2 Departments of Molecular and Medical Pharmacology, David Geffen School of Medicine at UCLA, University of California, Los Angeles, CA USA; 3 Department of Pediatrics, University of California, San Diego, La Jolla, CA USA; 4 Hebert Wertheim School of Public Health and Longevity Science, University of California, San Diego, La Jolla, CA USA; 5 Larsson-RosenquistFoundation Mother-Milk-Infant Center of Research Excellence (MOMI CORE), University of California, San Diego, La Jolla, CA USA

## Abstract

**Background:**

Genomic RNA of severe acute respiratory syndrome-associated coronavirus type 2 (SARS-CoV-2) has been detected in the breast milk of lactating women, but its pathological significance has remained uncertain due to the small size of prior studies.

**Methods:**

Breast milk from 110 lactating women was analyzed by reverse transcription-polymerase chain reaction (285 samples) and viral culture (160 samples). Those containing SARS-CoV-2 viral RNA (vRNA) were examined for the presence of subgenomic RNA (sgRNA), a putative marker of infectivity.

**Results:**

Sixty-five women had a positive SARS-CoV-2 diagnostic test, 9 had symptoms but negative diagnostic tests, and 36 symptomatic women were not tested. SARS-CoV-2 vRNA was detected in the milk of 7 (6%) women with either a confirmed infection or symptomatic illness, including 6 of 65 (9%) women with a positive SARS-CoV-2 diagnostic test. Infectious virus was not detected in any culture and none had detectable sgRNA. In control experiments, infectious SARS-CoV-2 could be cultured after addition to breastmilk despite several freeze–thaw cycles, as it occurs in the storage and usage of human milk.

**Conclusions:**

SARS-CoV-2 RNA can be found infrequently in the breastmilk after recent infection, but we found no evidence that breastmilk contains an infectious virus or that breastfeeding represents a risk factor for transmission of infection to infants.

**Impact:**

- This article goes beyond prior small studies to provide evidence that infectious SARS-CoV-2 is not present in the milk of lactating women with recent infection, even when SARS-CoV-2 RNA is detected.
- Recent SARS-CoV-2 infection or detection of its RNA in human milk is not a contraindication to breastfeeding.

## Introduction

To date, the coronavirus disease 2019 (COVID-19) pandemic has infected nearly 170 million people globally and over 33 million people in the United States.^1^ COVID-19 cases in infants represent about 50% of the total reported in young children globally,^2^ and infections of infants represent approximately 1.9% of all cases in the United States.^3^ Most newborns and infants fare well, but severe disease and death have been reported in newborns, infants, and young children.^2^ In addition, the multisystem inflammatory syndrome in children can occur even after apparent resolution of infection and is disproportionately affecting Black and Hispanic/Latino children.^4^ Understandably, there has been great concern about the potential consequences of severe acute respiratory syndrome-associated coronavirus type 2 (SARS-CoV-2) transmission to infants by postnatal exposures, including breastfeeding. There is limited but increasing evidence that breast milk is not a source of SARS-CoV-2 infection to infants who are breastfed by mothers who have evidence of recent infection.^5,6^ Therefore, the US Centers for Disease Control and Prevention, the American Academy of Pediatrics, and the World Health Organization advise that mothers who are infected with SARS-CoV-2 may choose to initiate or to continue breastfeeding an infant or toddler with appropriate protections to prevent transmission of the virus through respiratory droplets.^7,8^

We previously presented initial results from a study of 64 breast milk samples from 18 women with recent SARS-CoV-2 infection.^9^ A sample from one symptomatic woman was found to contain SARS-CoV-2 viral RNA (vRNA), but replication-competent virus was not detected in viral culture. In addition, we used viral culture methods to demonstrate that the conditions of Holder pasteurization inactivated SARS-CoV-2, a finding subsequently confirmed by others.^10–12^ To better examine the frequency and state of SARS-CoV-2 in the breast milk of women with recently documented infection, we present results from a larger observational cohort study. We also examined the vRNA found in a reverse transcription-polymerase chain reaction (RT-PCR)-positive samples for the presence of SARS-CoV-2 subgenomic transcripts, a proposed marker of viral infectivity.^13–15^ In addition, we examined the impact of breast milk on SARS-CoV-2 thermal stability to investigate the apparent discordance between viral culture and vRNA detection.

## Materials and methods

### Participants and breast milk specimens

As previously described,^9^ breast milk samples and clinical information were obtained from women participating in the Mommy’s Milk Human Milk Biorepository at the University of California, San Diego (IRB#130658). Between March 2020 and September 2020, we enrolled women residing in the United States who were symptomatic but not tested, symptomatic but with negative SARS-CoV-2 testing by RT-PCR, women exposed to an infected person, or those who had a confirmed SARS-CoV-2 infection by RT-PCR. Information about demographics, health history, illness and exposure dates, symptoms, and SARS-CoV-2 test results were collected by participant interview via telephone. Participants self-collected breast milk samples using a provided collection kit including instructions for expressing and storing their samples. The instructions provided specific instructions for hand washing before and after milk expression. Participants who had recovered from their illness at the time of the study interview were asked to ship any frozen samples previously collected at the peak of their symptoms in addition to a fresh milk sample. Fresh samples were shipped on ice within 24 h of collection to the Biorepository and stored at −80 °C prior to shipment on dry ice to the University of California, Los Angeles. This report includes samples from 18 women described in our previous report.^9^

### Virologic methods

The PCR-based methods used to detect SARS-CoV-2 RNA in skim milk and the culture techniques to detect replication-competent SARS-CoV-2 in whole breast milk have been previously described.^9^ The concentration of replication-competent virus in SARS-CoV-2 viral stocks was determined by limiting dilution culture in Vero-E6 (*Cercopithecus aethiops*-derived epithelial kidney) cells, calculated using the Spearman–Karber method, and expressed as the median tissue culture infectious doses/mL (TCID_50_/mL). RT-PCR analysis to detect subgenomic SARS-CoV-2 RNA (a proposed marker of viral replication^13–15^) was performed using oligonucleotide PCR primers WHSA-00025F and WHSA29925R^13^ and a Power Syber Green RNA to Ct 1-Step Kit (Thermo Fisher Scientific), following the manufacturer’s instructions.

### Thermal stability of SARS-CoV-2

We previously reported that SARS-CoV-2 could not be grown from a whole breast milk specimen that was shown to contain a high concentration of SARS-CoV-2 RNA.^9^ In that report, we also noted that the temperature conditions of Holder pasteurization rapidly inactivated live virus, even if diluted ten-fold in breast milk from healthy donors. However, it remained possible that we were unable to grow SARS-CoV-2 due to the presence of antimicrobial factors in breast milk that could reduce viral infectivity during the cycles of freezing and gradual low-temperature thawing that commonly occur with storage and preparation of breast milk. To examine the thermal stability of the virus during these steps, we added a small amount (100 TCID_50_) of SARS-CoV-2 (USA-WA1/2020) to four aliquots each of whole breast milk from two different healthy women. One aliquot from each set was then held at 4 °C, and three others were frozen again at −80 °C. These aliquots were slowly thawed to room temperature (approximately 20 °C). Two aliquots were frozen again to −80 °C, and one was held at 4 °C. This process was repeated, yielding samples that had undergone two freeze–thaw cycles. Following a third freeze and thaw for aliquots from both women, all samples were stored at 4 °C for 3 days. Using previously described methods,^9^ we inoculated viral cultures with this spiked milk and examined them for cytopathic effect (normally easily identified by 4 days of culture). After 4 days of culture, RNA was extracted from the culture supernatants and RT-PCR^9^ was used to detect SARS-CoV-2 genomic RNA.

### Statistical analysis

Maternal and infant characteristics were compared between the group of mothers with confirmed infection for whom no milk samples were positive and those with either confirmed infection or who were symptomatic and who had at least one milk sample that was positive for vRNA. Comparisons for continuous variables were made using Wilcoxon’s rank-sum test. Categorical variables were compared using Fisher’s exact test. Missing values were excluded. SPSS version 25 was used for analyses, and Prism version 8.4.3 (GraphPad) was used for figure presentation.

## Results

### Detection of SARS-CoV-2 RNA in the breast milk of women

Breast milk samples were available from 110 women: 36 women with SARS-CoV-2 symptoms, but no diagnostic test, 9 women with symptoms but a negative nasal or nasopharyngeal SARS-CoV-2 RT-PCR test, and 65 study participants with confirmed SARS-CoV-2 using a nasal or nasopharyngeal RT-PCR test (including 18 previously described women^9^). A total of 336 breast milk samples were submitted by these participants (median of 2.5 samples of breast milk/woman (range 1–13). Out of 336 samples, 285 breast milk samples (85%) were available and analyzed for SARS-CoV-2 vRNA (six unsuccessful PCR assays). A total of 160 samples were cultured for the virus: 118 from 50 of the women with positive SARS-CoV-2 nasopharygeal PCR tests and 42 from the two groups of symptomatic women. All subsequent descriptors presented below are restricted to the 65 women who had a confirmed diagnosis of SARS-CoV-2 and 1 asymptomatic woman whose milk sample was positive for vRNA, but who was not tested for SARS-CoV-2. We will refer to this group as the 66 women with confirmed SARS-CoV-2 infection.

The majority of women were non-Hispanic and white with a median age of 35.8 years (Table 1). Among these, 59 (89%) had symptomatic COVID-19. Only 6 (9.1%) were hospitalized, and of these, two developed respiratory failure and required extracorporeal membranous oxygenation (ECMO) support. Two-thirds (66.7%) of the women who were hospitalized were pregnant at the time of their SARS-CoV-2 infection and hospitalizations. The two women with severe disease requiring ECMO ultimately delivered their infants while receiving treatment.

**Table 1:** Characteristics of 66 women with confirmed SARS-CoV-2 infection^a^.

	No detectable SARS-CoV-2 in breast milk samples, *n* (%), *N* = 59	Detectable SARS-CoV-2 in breast milk samples, *n* (%), *N* = 7	*P* value
Maternal age (median years [IQR])	35.80 [25.56, 46.67]	32.28 [27.62, 38.66]	0.120
Race			1.000
Caucasian	48 (84.21)	7 (100.00)	
Black	1 (1.75)	0 (0.00)	
Asian	5 (8.77)	0 (0.00)	
Native American/Alaska native	2 (3.51)	0 (0.00)	
Multiracial	1 (1.75)	0 (0.00)	
Ethnicity			0.528
Hispanic	5 (8.77)	1 (14.29)	
Non-Hispanic	52 (91.23)	6 (85.71)	
US region of residence			0.019
North-East	21 (35.59)	1 (14.29)	
South	15 (25.42)	3 (42.86)	
Mid-West	6 (10.17)	3 (42.86)	
West	17 (28.81)	0 (0.00)	
BMI ≥ 30	20 (35.08)	0 (0.00)	0.096
Underlying health condition^b^	15 (26.32)	2 (28.57)	1.000
Symptomatic	53 (89.83)	6 (100.00)	1.000
Hospitalized	6 (10.71)	0 (0.00)	1.000
Treatment received: Remdesivir	3 (5.26)	0 (0.00)	1.000
Treatment received: ECMO	2 (3.51)	0 (0.00)	1.000
Source of infection			0.517
Household	17 (29.31)	3 (42.86)	
Community	11 (18.97)	2 (28.57)	
Occupational	18 (31.03)	2 (28.57)	
Unknown	12 (20.69)	0 (0.00)	
Infant age (median months [IQR])	7.97 [0.10, 35.28]	9.63 [2.79, 15.65]	0.498
Infant sex			0.691
Male	25 (43.86)	4 (57.14)	
Female	32 (56.14)	3 (42.86)	

^a^Sixty-five who tested positive by RT-PCR of the nasopharyngeal specimen and 1 without diagnostic testing whose breast milk sample was positive by RT-PCR.

^b^Underlying medical conditions included the following: asthma, diabetes type II, heart defect, hypertension, hypothyroidism, irritable bowel disease, kidney defect, obesity, and “tachycardia”.

**Fig. 1:**
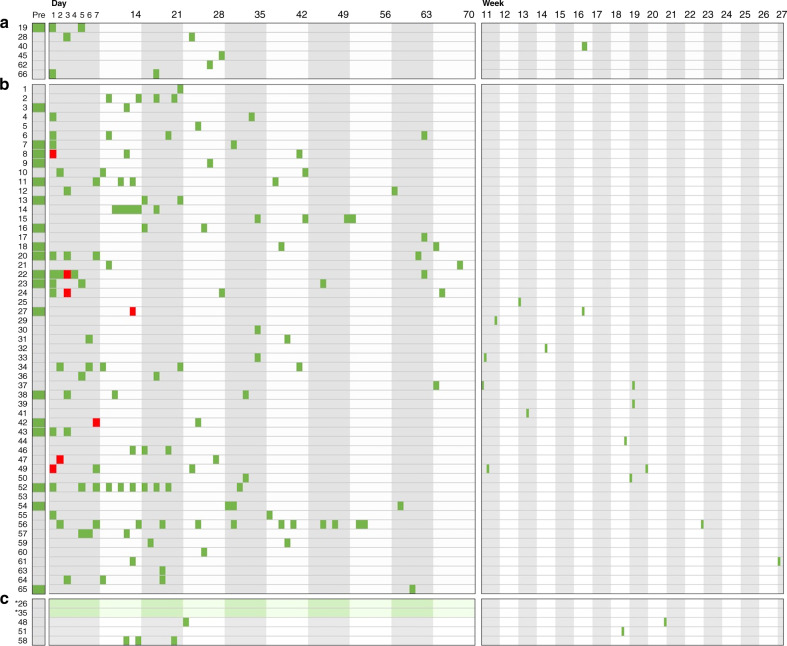
Sample collection timing by day/week from the onset of symptoms or hospitalization for 65 women who tested positive for SARS-CoV-2 infection and 1 woman who was symptomatic but untested. Panel **a** displays the timing of breast milk samples collected from women who lacked any symptoms of COVID-19 (SARS-CoV-2 infection). Panel **b** displays the timing of breast milk samples collected by symptomatic women who were not hospitalized. Panel **c** displays are those who were symptomatic and hospitalized. Each colored rectangle demonstrates the time of collection of breast milk specimens in relation to their onset of SARS-CoV-2 symptoms or for those women who were asymptomatic, in relation to their positive test date. Red squares indicate specimens in which the RNA of SARS-CoV-2 was detected by RT-PCR. Asterisks indicate the two women (#26 and #35) who required ECMO support; the date of collection is not precisely known and green shading is therefore used to indicate the period during which the breast milk was collected.

SARS-CoV-2 viral RNA was detectable in one milk sample each from 7 of the 66 with confirmed infection (Fig. 1). The women with detectable SARS-CoV-2 RNA in their breast milk did not differ in age, race or ethnicity, or other demographic parameters from women with negative breast milk samples (Table 1). For the seven women whose milk samples contained viral RNA, SARS-CoV-2 RNA was not detected in the next specimen of milk available, ranging from 1 to 97 days later (Table 2 and Fig. 1). There was no clinical evidence of infection among any of the infants being breastfed by the seven women with documented SARS-CoV-2 RNA in breast milk.

**Table 2:** Summary of virologic data from breast milk samples with detectable SARS-CoV-2 RNA.

Participant	Number of symptoms^a^	Number of days symptomatic	Symptomatic at time of sample collection	SARS-CoV-2 RNA in milk samples (copies/mL)	sgRNA	Viral culture
8^b^	9	18	Yes	25,100	Negative	Negative
22	10	17	Yes	2400	Negative	Negative
24	16	65	Yes	3230	Negative	Negative
27^c^	9	13	Yes	10,000	Negative	Negative
42	8	17	Yes	12,000	Negative	Negative
47	5	4	Yes	11,200	Negative	Negative
49	7	7	Yes	150	Negative	Negative

^a^See Supplemental information (Table S1) for a list of COVID-19-related symptoms.

^b^Previously reported.

^c^Woman was symptomatic but not tested.

### Cultures and subgenomic RT-PCR reveal no evidence of infectious SARS-CoV-2 in breast milk

None of the viral cultures of 160 breast milk specimens was positive. We used a second real-time RT-PCR assay to further analyze the seven milk specimens found to contain vRNA for the presence of subgenomic RNA (sgRNA) (Table 2). sgRNAs detected by this assay are spliced RNAs that are produced during coronavirus replication.^15^ The milk specimens were clarified by centrifugation and RNA was extracted from the skimmed milk as before. sgRNA was not detected in any of these seven specimens.

### Stability of SARS-CoV-2 in human milk

We considered the possibility that freezing and thawing of breast milk inactivates SARS-CoV-2 and prevents its detection by culture. We, therefore, added a small inoculum (100 TCID_50_) of infectious SARS-CoC-2 to milk samples from two healthy women, performed serial freeze–thaw cycles, and then stored the samples at 4 °C for 3 days. After this prolonged storage, we added the milk to viral cultures. After 4 days of culture, we removed culture supernatants and used RT-PCR to confirm the presence of viral RNA. Samples that had undergone as many as three freeze and thaw cycles continued to exhibit high infectivity: cytopathic effect spread throughout the cultures within 3 days (just as quickly as positive control wells inoculated with fresh SARS-CoV-2 alone). These experiments demonstrated that the temperature excursions that frequently occur during the handling of breast milk would not prevent us from detecting infectious SARS-CoV-2.

## Discussion

Breast milk is an invaluable source of nutrition to infants^16,17^ and contains numerous antimicrobial factors, including neutralizing antibodies to viruses.^18,19^ Of note, antibodies to SARS-CoV-2 have been detected in the breast milk of recently infected lactating women.^20–22^ However, breastfeeding is clearly a route by which either HIV or HTLV (human T cell lymphotropic virus) can be transmitted to infants. In contrast to these pathogens, hepatitis B virus and hepatitis C virus infections produce chronic infections in women and yet are not contraindications to breastfeeding.^23^

A few small case reports have described the detection of SARS-CoV-2 RNA in breast milk^5,24^ and one report suggested the possibility of vertical transmission, although contamination could not be ruled out as the source.^25^ Prior reports have generally examined the breast milk of infected mothers for the presence of the virus using RT-PCR methods.^9,25–33^ In one case, SARS-CoV-2 was said to be detectable by RT-PCR in four milk samples collected between 6 and 10 days after the mother’s first positive test. The authors indicated there was no evidence that contamination of the milk samples could have been the source of the virus. However, these reports do not give an overall estimate of the frequency of viral RNA and infectious SARS-CoV-2 in human milk, and the report of possible transmission did not exclude the possibility of contamination of milk occurring at the time of collection.^24^ The rationale for this study was to determine how often SARS- CoV-2 viral RNA was present in breast milk samples and to examine the risk of infection to infants through breast milk. This study is unprecedented in the use of viral cultures to examine a very large number of breast milk specimens, a limitation of prior studies cited by Lackey et al.^24^

We found that SARS-CoV-2 RNA is seldom detected in breast milk samples from women with confirmed SARS-CoV-2 infection. Moreover, our longitudinal follow-up indicates that even when it is detected, it is an unlikely source of infection for the breastfed baby: viral RNA was only transiently present and we were unable to culture SARS-CoV-2 from any sample.

This study had several limitations as well as strengths. The collection of breast milk samples was not directly observed and we relied on the maternal report of SARS-CoV-2 test results, symptoms, and treatments received. However, all participants completed a semistructured interview guided by trained study staff who prompted for specifics with the aid of a calendar. In addition, to our knowledge, this study represents the largest number of breast milk samples analyzed to date from women with recent SARS-CoV-2 infection. Nonetheless, even this size may have been too small to permit the identification of factors that would predict the presence of SARS-CoV-2 RNA in breast milk.

We demonstrated that the SARS-CoV-2 maintains its infectivity despite repeated freezing and thawing and storage at 4 °C. While sgRNA (an indicator of virus replication) was not detected in any of the milk specimens already known to contain SARS-CoV-2 RNA, this assay is only moderately sensitive compared to PCR detection of genomic RNA. For example, sgRNA is present in only about half of nasopharyngeal specimens with positive viral cultures (see refs. ^13,15^ and unpublished data by P.K.).

## Conclusion

These data provide substantial evidence that breastfeeding from women proven or suspected to have had SARS-CoV-2 infection does not represent a hazard for infants.

## Supplementary information

Supplementary Material
