# Supplementary material for: No infectious SARS-CoV-2 in breast milk from a cohort of 110 lactating women
Source: Pediatr Res. 2022 Jan 19;92(4):1140–5. doi: 10.1038/s41390-021-01902-y (PMC9586866; doi:10.1038/s41390-021-01902-y)
Supplement: Supplementary file 1 — Supplementary Material [file 41390_2021_1902_MOESM1_ESM.docx]

Supplemental information to accompany Krogstad et al (2021).

**Table S1: List of COVID-19 related symptoms used during initial interview of study participants.**


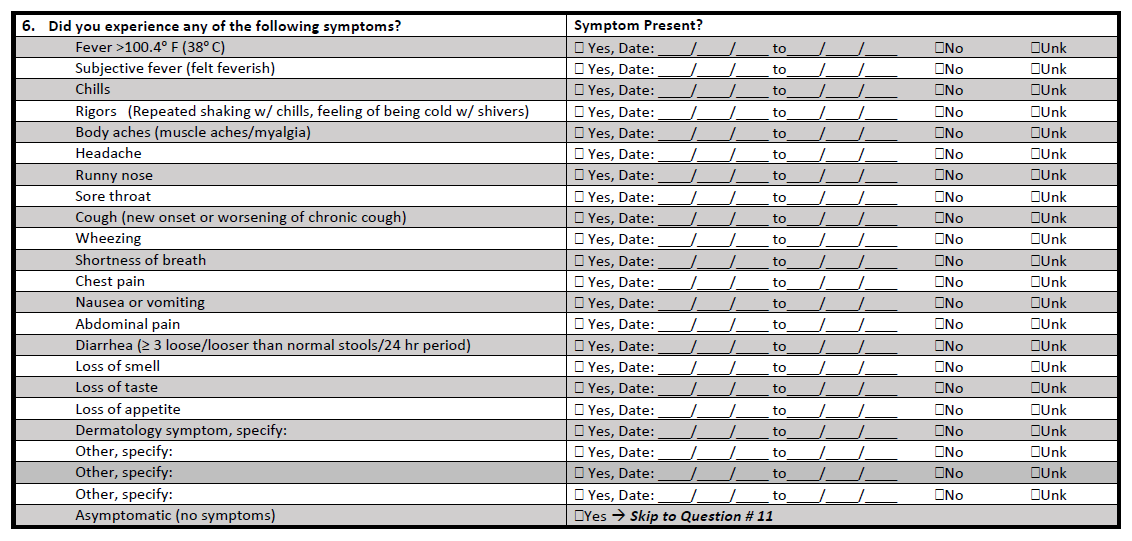


For example: Participant #27 was symptomatic and reported:

- Subjective Fever (felt feverish)

- Chills

- Headache

- Runny Nose

- Sore Throat

- Cough

- Wheezing

- Diarrhea

- Loss of appetite
